# Machine learning techniques for the identification of risk factors associated with food insecurity among adults in Arab countries during the COVID-19 pandemic

**DOI:** 10.1186/s12889-023-16694-5

**Published:** 2023-09-16

**Authors:** Radwan Qasrawi, Maha Hoteit, Reema Tayyem, Khlood Bookari, Haleama Al Sabbah, Iman Kamel, Somaia Dashti, Sabika Allehdan, Hiba Bawadi, Mostafa Waly, Mohammed O. Ibrahim, The Regional CORONA COOKING Survey Group, Charlotte De Backer, Lauranna Teunissen, Kathleen Van Royen, Isabelle Cuykx, Paulien Decorte, Gaëlle Ouvrein, Karolien Poels, Heidi Vandebosch, Katrien Maldoy, Sara Pabian, Christophe Matthys, Tim Smits, Jules Vrinten, Ann DeSmet, Nelleke Teughels, Maggie Geuens, Iris Vermeir, Viktor Proesmans, Liselot Hudders, Mariam Al-Mannai, Tariq Alalwan, Elissa Naim, Rania Mansour, Nour Yazbeck, Hazem Agha, Rania Abu Seir, Jamila Arrish, Ghadir Fallata, Omar Alhumaidan, Shihana Alakeel, Norah AlBuayjan, Sarah Alkhunein, Budur Binobaydan, Aeshah Alshaya, Ayesha Aldhaheri, Stephanny Vicuna Polo, Diala Abu Al-Halawa

**Affiliations:** 1 Department of Computer Science, Al-Quds University, Jerusalem, Palestine; 2 Department of Computer Engineering, Istinye University, Istanbul, 34010 Turkey; 3 Faculty of Public Health, Lebanese University, Beirut, Lebanon; 4 PHENOL Research Group (Public Health Nutrition Program Lebanon), Faculty of Public Health, Lebanese University, Beirut, Lebanon; 5 Lebanese University Nutrition Surveillance Center (LUNSC), Lebanese Food Drugs and Chemical Administrations, Lebanese University, Beirut, Lebanon; 6 Department of Human Nutrition, College of Health Sciences, QU-Health, Qatar University, Doha, Qatar; 7 Department of Nutrition and Food Technology, Faculty of Agriculture, University of Jordan, Amman, 11942 Jordan; 8 National Nutrition Committee, Saudi Food and Drug Authority, Riyadh, Saudi Arabia; 9 Department of Clinical Nutrition, Faculty of Applied Medical Sciences, Taibah University, Madinah, Saudi Arabia; 10 Department of Health Sciences, College of Natural and Health Sciences, Zayed University, Dubai, United Arab Emirates; 11 National Research Centre, Cairo, Egypt; 12 Public Authority for Applied Education and Training, Kuwait City, Kuwait; 13 Department of Biology, College of Science, University of Bahrain, Zallaq, Bahrain; 14 Food Science and Nutrition Department, College of Agricultural and Marine Sciences, Sultan Qaboos University, Muscat, Oman; 15 Department of Nutrition and Food Technology, Faculty of Agriculture, Mu’tah University, Karak, Jordan; 16 Department of Faculty of Medicine, Al Quds University, Jerusalem, Palestine

**Keywords:** Food insecurity, COVID-19, Food consumption score, Machine learning, Prediction, Arab countries

## Abstract

**Background:**

A direct consequence of global warming, and strongly correlated with poor physical and mental health, food insecurity is a rising global concern associated with low dietary intake. The Coronavirus pandemic has further aggravated food insecurity among vulnerable communities, and thus has sparked the global conversation of equal food access, food distribution, and improvement of food support programs. This research was designed to identify the key features associated with food insecurity during the COVID-19 pandemic using Machine learning techniques. Seven machine learning algorithms were used in the model, which used a dataset of 32 features. The model was designed to predict food insecurity across ten Arab countries in the Gulf and Mediterranean regions. A total of 13,443 participants were extracted from the international Corona Cooking Survey conducted by 38 different countries during the COVID -19 pandemic.

**Results:**

The findings indicate that Jordanian, Palestinian, Lebanese, and Saudi Arabian respondents reported the highest rates of food insecurity in the region (15.4%, 13.7%, 13.7% and 11.3% respectively). On the other hand, Oman and Bahrain reported the lowest rates (5.4% and 5.5% respectively). Our model obtained accuracy levels of 70%-82% in all algorithms. Gradient Boosting and Random Forest techniques had the highest performance levels in predicting food insecurity (82% and 80% respectively). Place of residence, age, financial instability, difficulties in accessing food, and depression were found to be the most relevant features associated with food insecurity.

**Conclusions:**

The ML algorithms seem to be an effective method in early detection and prediction of food insecurity and can profoundly aid policymaking. The integration of ML approaches in public health strategies could potentially improve the development of targeted and effective interventions to combat food insecurity in these regions and globally.

## Introduction

Food insecurity (FI) has been a major global health concern, particularly in low- and middle-income countries. FI is defined as a lack of access to sufficient and affordable food [1, 2]. Improper diet, unhealthy eating habits and lifestyles [2–4], limited food availability, lack of access to food, and proper food consumption to meet dietary needs have been evidenced as major factors that contribute to food insecurity [5–7]. According to the State of Food Security and Nutrition in the World (SOFI) report, food insecurity can have a serious impact on people’s health of all ages and may include several adverse health effects such as depression, diabetes, obesity, anxiety, and hypertension [8]. Furthermore, the SOFI report indicated that the COVID-19 pandemic increased chronic hunger between 2019 and 2020 [8]. The Global Humanitarian Overview report indicated that about 265 million people needed humanitarian assistance during the year 2021 due to the COVID-19 pandemic [9–11]. Several studies reported that the COVID-19 pandemic significantly impacted global food insecurity and poverty, mainly in low- and middle-income countries that have limited access to food, as they experienced high rates of unemployment, income inequality, and disruption of social safety programs [11, 12].

In the Arab region, mainly in Jordan, Lebanon and Palestine, the pandemic has severely affected the countries’ national economies, in particular the services sectors, the supply chain, the markets, and trade, all of which have directly impacted food supply, demand, and access [8, 11]. Several studies reported that low- and middle-income countries were most affected by the COVID-19 pandemic, and thus presented an increase in food insecurity [6, 10]. A United Nations Economic and Social Commission for Western Asia (ESCWA) study reported that around 8.3 million people in the Arab region ran the risk of falling into poverty and income crises due to lockdowns, which increased the number of food-insecure and undernourished persons [5, 10]. Furthermore, Kharroubi et al.’s study indicated that food insecurity among Lebanese increased from 27% to 36%-39% due to the pandemic [5, 10]. Similarly, World Food Program (WFP) reports indicated that 53% and 34% of Jordanians and Palestinians are vulnerable to food insecurity respectively [1, 9, 13, 14]. Although Gulf Cooperation Council (GCC) countries (Bahrain, Kuwait, Oman, Qatar, Saudi Arabia, and United Arab Emirates) have been considered the most food-secure countries in the Arab world, the COVID-19 pandemic and the current global crises have affected food accessibility and caused food insecurity concerns in the GCC region [15, 16].

Food insecurity is an interdimensional concept that encompasses food availability, access to food, and food consumption diversity. Food insecurity is prevalent when individuals are limited or are unable to acquire nutritious food without resorting to emergency situations. Food security, on the other hand, represents a condition whereby a person has “physical and economic access to sufficient, safe, and nutritious food that meets their dietary preference for an active and healthy lifestyle” [17]. Several traditional methods have been developed for the assessment and depiction of households’ food insecurity. However, current frameworks for identifying food in/security do not incorporate statistical models that account for data on key indicators. Indeed, the status quo for identifying food insecurity and its risk factors is based on international food assessments. For instance, the Food Security Phase Classification System (IPC) presents key challenges in terms of early warning for food insecurity as they are infrequent and complex, its assessments are difficult to replicate or confirm, and does not integrate a full scope of data that integrates all variables [18].

In addition, the Food Consumption Score (FCS) has been used to measure the quantity and diversity of food consumption [17–19]. Furthermore, household surveys, satellite-based remote sensing, the FAO’s Food Balance Sheets, and vulnerability analyses, among others are also utilized for analyzing food security. Although these methods offer insights into food security dynamics, there is a lack of consensus about preferred methodologies and reflects inefficient results of survey instruments when collecting information on food insecurity from several dimensions [19]. In fact, no existing survey instrument is currently able to collect all necessary indicators in a timely manner, and with high frequency. Nonetheless, this paper aims to demonstrate a novel model utilizing machine learning for the identification of food in/security.

Recently, machine learning (ML) techniques have introduced a transformative approach to this domain. It processes diverse datasets, identifies patterns, and predicts food shortages with enhanced accuracy [20–23]. ML’s iterative learning model, driven by continuous data integration, allows it to adjust predictions based on dynamic factors like climatic variances, geopolitical changes, and economic fluctuations. This adaptability ensures that food security interventions are data-driven and optimized. While traditional methods provide foundational understanding of food security challenges, machine learning strengthens this knowledge, offering a more robust and responsive toolset for addressing the multifaceted and ever-evolving challenges of food security.

Data mining and Machine Learning (ML) techniques have been used in several studies as efficient tools for predicting and identifying the risk factors associated with food insecurity [20, 24–26]. K-Nearest Neighbor (KNN), Random Forest (RF), Logistic regression and Support Vector Machine (SVM) are among the ML models that have been assessed for the prediction of food insecurity [24, 27, 28]. A number of studies utilizing ML techniques have been pivotal by introducing innovative and comprehensive methodologies for data classification and variable identification. In rural Bangladesh, Hossain et al. attempted to determine food insecurity by studying the effectiveness of integrating various household indicators. Their methodological design aimed at obtaining accurate and budget-friendly indicators that could serve as reliable proxies for calorie intake, a pivotal metric in food security. They used a comparative approach, introducing subjective indicators and dietary diversity to an initial basic dataset. The inclusion of these additional indicators elevated the predictive accuracy significantly from 63% to 69%, emphasizing the value of multifaceted data inputs [29].

Similarly, Meerza et al. study aimed to identify key household characteristics that distinguish between food-secure (FS) and food-insecure (FI) households in Bangladesh. The research balanced both traditional statistical methods and advanced machine learning models. Among the identified predictors, factors such as household size, household expenditure, livestock assets, age of the household head, and household assets. The proposed model achieved an 83 percent accuracy in predicting FI households within the sample. The use of interpretable ML techniques also allowed for understanding the nature of the association between FI households and household characteristics [28]. Gao et al., in their study on Afghan households, examined the ML models to identify vulnerable household characteristics and to predict food-insecure households [24]. Their analytical framework not only confirmed the relevance of well-established determinants, such as income levels and household size, but also brought to the fore unconventional indicators, like the material composition of dwelling walls, indicative of accumulated wealth. This approach advances the limits of traditional thought, suggesting the multi-dimensional nature of factors influencing food security [24].

Moreover, a variety of ML techniques have been assessed by researchers, some have produced higher prediction scores. For instance, Christensen et al. involved the application of machine learning in predicting food crises in multiple countries using a dataset from the World Bank. Their ambitious approach exceeded traditional models, leveraging the power of neural networks. The analysis revealed a positive correlation between food crises and the vegetation index and food price index. The year-agnostic neural network model showed excellent performance, achieving high recall, precision, and f1 scores, surpassing current prediction efforts for food crises [30].

Furthermore, In Georgiana et al.’s study, the ML models were used for identifying food insecurity in the food sharing network, the authors indicated that Random Forest and AdaBoost had higher prediction accuracies and produced a complex features structure that contributes to food insecurity [31]. In Doreswamy et al.’s study, the ML models were used in household food insecurity classifications, among them are the KNN, Logistic Regression (LR), RF, and SVM models; the RF reported the best accuracy for features classifications and importance with an accuracy rate of 99.98% [32]. The main objective of the study was to develop a predictive model that assists in the early identification of food insecurity, rather than to directly reverse the adverse effects of food insecurity. The features that are affected by food insecurity, like BMI or certain lifestyle habits, were included in the model as potential predictors, given their likely role in identifying the risk of food insecurity. Despite their mutual relationship with food insecurity, these factors’ inclusion can potentially improve the model’s performance in detecting those at earlier risk.

The reasons of including these features in the ML model in the study based on the assumption that many aspects of an individual’s life, including their health status (like BMI), lifestyle habits, and social circumstances, can act as indicators of their food security status [33, 34]. For example, an individual with a low BMI might be undernourished due to limited access to sufficient food, suggesting a potential risk of food insecurity [30]. Conversely, if an individual is food insecure, they may not have access to sufficient or nutritious food, leading to a lower BMI. This complex relationship is similarly observed with lifestyle habits. Certain habits, such as infrequent meal patterns or reliance on less nutritious food, could be both a reaction to and a predictor of food insecurity.

The ML model used in this study for identifying patterns and making accurate predictions, doesn’t necessarily elucidate causality. The features used in the model could be viewed as indicators that can assist in identifying individuals or communities potentially at risk, rather than definitive causes of food insecurity. However, it is crucial to note that ML should complement other types of analysis and human judgment in shaping interventions and policies. Therefore, a more comprehensive understanding of the cause-and-effect dynamics in food insecurity would impose further research and a multi-disciplinary approach to fully capture the issue’s complexity.

The existing studies have made significant contributions to understanding the use of ML models in predicting and identifying risk factors associated with food insecurity, nevertheless, the existing models mainly focused on food accessibility and availability. However, a knowledge gap still exists in providing valuable insights into the quality and diversity of food available to the targeted population, which are key elements of food security, in addition to the inclusion of other factors, such as socio-economic and socio-demographic variables. While the COVID-19 pandemic’s impact on food insecurity is discussed, the preparedness of these models to predict the outcomes of future unforeseen global or regional pandemics remains unaddressed. Furthermore, the studies don’t sufficiently consider the need for localized models given cultural and economic disparities, as they mainly focus on individual factors over broader societal dynamics, the translation of findings into policies, the long-term implications of ML interventions, and capacity building in areas with limited resources. Nonetheless, the literature provides valuable insights into the state of food insecurity and the potential of ML in predicting it. Yet, there is a clear need for more comprehensive research that bridges the gaps identified, offering a holistic approach to the challenge of food insecurity.

Consequently, this study provides better understanding of the direct consequences of the COVID-19 pandemic on health and poverty indices in low- and middle-income countries. Therefore, this study aims to investigate the performance of different ML techniques in predicting and identifying risk factors associated with food insecurity based on food consumption score, which represents the food quality and diversity during the COVID-19 pandemic, specifically focusing on the impact of the pandemic on health and poverty indices in the region.

## Methods

### Aim

The study aimed to identify the key determinants of food insecurity within the Arab region during the COVID-19 pandemic through a novel approach, ML algorithms.

### Setting

The “Corona Cooking Survey April 2020” [35] conducted by 38 different countries during the COVID -19 pandemic was used for this study. The survey was distributed to over 300 participants per country in each of the following Arab countries: (Palestine, Lebanon, Jordan, Kuwait, Oman, Qatar, Saudi Arabia, United Arab Emirates, and Bahrain. The survey data was collected by the research team between April 17^th^ and June 25^th^, 2020.

### Design

#### Data source

The dataset was extracted from the international “Corona Cooking Survey April 2020” [29]. The survey was designed as a multi-language survey including the Arabic language to facilitate data collection among different countries. The data collection instrument assessed the effect of the COVID-19 lockdown on adults’ health and nutrition. The survey included several types of information, such as COVID-19 Lockdown Measures, COVID-19 Feelings (Kessler K6 scale), Food Literacy Scale, Shopping Experiences and Behavior, Cooking Behavior and Attitudes, Seeking Recipes & Food Content, Eating Behaviors (Food Frequency Questionnaire), Food Advice Sources, E-drinking and E-dining, anthropometric measurements, and lifestyle and eating habits. The survey was distributed through different social media platforms and through the partner universities’ networks. The final sample included over 300 participants per country. Countries that acquired a lesser number of samples were excluded from the analysis. The data relating to food insecurity in Arab countries were extracted from the International Survey. Overall, 13,443 participants aged over 18 years were included in the ML model development.

#### Model features

The features were extracted from the main study variables, accessible via https://osf.io/nz9xf/files/ [29]. The study features contain respondents’ data from before and after the COVID-19 lockdown. The ML models used food insecurity as the main target variable for assessing the performance of ML in predicting and identifying associated risk factors. Food insecurity was defined in reference to the Food Consumption Score (FCS), which is an indicator used to assess dietary diversity before and after the pandemic. The FCS was categorized into two groups: Low FCS if the FCS < 42 (Unacceptable), and high FCS score if the FCS scores >  = 42 (Acceptable). Thus, food insecurity was determined based on the FCS classification. The low FCS group was considered a food insecure group, while the high FCS group was considered a normal group. Detailed information about the study variables’ definition and calculation can be found in Hoteit et al. [29].

The study used interconnected nature of social determinants of health, clinical features, and food insecurity. By understanding these relationships, the study attempts to develop an early detection system for food insecurity using ML models.

The social determinants of health, which include factors like socioeconomic status, education, and physical environment, among others, have been well-documented to influence access to sufficient and nutritious food [9, 31]. For instance, low-income households might struggle to afford healthy meals, contributing to food insecurity. On the contrary, communities with food insecurity may face limited educational and economic opportunities, thus creating a feedback loop. By identifying such patterns, the ML model can predict potential food insecurity based on existing social determinants of health [32, 36]. As for clinical features, factors such as body mass index (BMI) or presence of certain health conditions may provide valuable insights into an individual’s nutritional status, which is intrinsically linked to food security [10]. For instance, an unusually low BMI might suggest malnutrition due to inadequate food access, a possible sign of food insecurity [37]. Meanwhile, certain diseases may impact a person’s ability to obtain or consume nutritious food, thereby exacerbating food insecurity.

In our study the clinical features and social determinants of health were used as predictors of food insecurity, the existence of these factors provides information that an individual or a community is at risk. Therefore, by training ML models to recognize these features, we can potentially identify food insecurity early, even before more severe symptoms manifest. This enables timely interventions to alleviate food insecurity and prevent its harmful effects on physical and mental health.

#### Data preprocessing

Data underwent a preprocessing procedure prior to building the ML models. The preprocessing phase included data cleaning, formatting, missing data treatment, and data categorization. The data cleaning process included the null value data, the text-to-numeric conversion, and the missing data treatment.

In the data set, imbalanced data was encountered as 1529 participants were categorized as food insecure, and 11,914 participants were categorized as food secure. An imbalanced data set might bias the ML model’s estimation by providing more weight to the dominant class [38]. The simple and effective Min–Max Normalization technique was used to scale features to a common range to ensure that all the features were on the same scale, and to allow the model to make more accurate predictions. The minimum and maximum values for each feature were first calculated, followed by subtracting the minimum value from all feature values and dividing the resulting tables by the range (i.e., the difference between the maximum and minimum values) to ensure that all the features were scaled between 0 and 1.

#### ML models description

Seven ML models were used in this study to assess the performance of ML in predicting food insecurity among Arab countries following the COVID-19 pandemic. Logistic Regression (LR), Gradient Boosting (GB), Support Vector Machine (SVM), Random Forest (RF), Artificial Neural Network (ANN), Naïve Bayes (NB), and k-nearest neighbors’ algorithm (k-NN) were built and evaluated considering their performance measures.

##### Logistic Regression (LR)

Logistic regression serves as a supervised machine learning algorithm for classification, facilitating the prediction of the likelihood of an outcome variable [23]. This outcome, or dependent variable, inherently has a binary nature, signifying that only two potential outcomes are possible. The algorithm’s extensive usage arises from its ability to construct a model reflecting the association between the dependent and independent variables by utilizing the logistic function. Essentially, logistic regression is a linear model that computes the likelihood of an observation being part of a specific class. The algorithm develops a linear equation aligned with the gathered data, subsequently applying a sigmoid function to convert the resultant value into a probability range of 0 to 1. This approach is rooted in the maximum likelihood estimation principle that aims to determine the best-fitting values for the coefficients tied to the independent variables. Regularization strength, denoted as (C), is a parameter that balances the perfect fit to the training data and mitigates overfitting. Another parameter, the penalty (L1 or L2), decides the type of regularization applied, where L1 encourages sparsity while L2 governs the size of the coefficients.

One of the appealing features of logistic regression is its ability to be interpreted. The coefficients tied to the independent variables depict both the extent and the direction of their impact on the dependent variable. The algorithm performs optimally when the association between independent variables and the dependent variable is linear or can be adjusted into a linear form. However, its effectiveness may decrease when handling intricate non-linear relationships.

##### Gradient Boosting (GB)

Gradient Boosting is a supervised machine learning algorithm used for classification and regression problems. It is a powerful boosting algorithm that combines several weak learners into strong learners, in which each new model is trained to minimize the loss function such as mean squared error or cross-entropy of the previous model using gradient descent. In each iteration, the algorithm computes the gradient of the loss function with respect to the predictions of the current ensemble and then trains a new weak model to minimize this gradient. The predictions of the new model are then added to the ensemble, and the process is repeated until a stopping criterion is met.

Gradient Boosting is an ensemble learning method that combines multiple weak learners, usually decision trees, to create a strong predictive model. GB builds the model in an iterative manner, focusing on the samples with higher errors in each iteration. It updates the model by adding weak learners that correct the mistakes of the previous ones [39].

The GB algorithm works by fitting the weak learners sequentially, where each subsequent learner learns from the mistakes of its predecessors. It assigns higher weights to the misclassified instances, thereby boosting their importance in subsequent iterations. The learning rate (learning_rate) determines the contribution of each weak learner to the final prediction.

GB has the advantage of being able to capture complex relationships between the features and the target variable. It handles high-dimensional data well and can effectively handle non-linear relationships. The maximum depth of individual regression estimators (max_depth) controls the complexity of the trees, while the number of boosting stages (n_estimators) determines the number of weak learners to be combined.

##### Support Vector Machine (SVM)

Support Vector Machine is a powerful supervised learning algorithm used for classification and regression tasks. SVM constructs a hyperplane or a set of hyperplanes in a high-dimensional space to maximize the separation between different classes [23, 39]. The choice of the kernel determines the decision boundary shape.

SVM works well when the classes are well-separated, and the number of features is relatively small. The kernel trick allows SVM to implicitly map the data into a higher-dimensional space, where the classes can be linearly separated. The regularization parameter (C) controls the trade-off between maximizing the margin and minimizing the classification error.

SVM aims to find the optimal hyperplane that maximizes the margin between the support vectors, which are the closest instances to the decision boundary. The kernel coefficient (gamma) controls the influence of individual training samples on the decision boundary. A low gamma value results in a smooth decision boundary, while a high gamma value makes the decision boundary more flexible.

##### Random Forest (RF)

Random Forest is an ensemble learning method that constructs multiple decision trees and combines their predictions to make a final decision. Each tree is built using a random subset of the training data and a random subset of the features [23, 39]. RF reduces overfitting and improves accuracy by averaging the predictions of multiple trees.

RF handles high-dimensional data well and can capture non-linear relationships between features and the target variable. The number of trees in the forest (n_estimators) determines the number of decision trees to be constructed. The maximum depth of the tree (max_depth) controls the depth of each individual tree, while the maximum number of features considered for splitting (max_features) determines the number of features to consider for each split. The criterion for splitting (gini or entropy) determines the measure of impurity used to evaluate the quality of a split.

Random Forest combines the predictions of the individual trees by either majority voting (classification) or averaging (regression). This ensemble approach helps reduce the impact of individual trees’ biases and improves the overall accuracy and robustness of the model.

One advantage of Random Forest is its ability to handle noisy data and outliers. It can also provide insights into feature importance, as it calculates the average decrease in impurity for each feature across all trees. Furthermore, it is computationally efficient and can handle large datasets with high-dimensional features.

##### Artificial Neural Network (ANN)

Artificial Neural Network is an adaptable and powerful ML model inspired by the structure and function of the human brain. ANN consists of interconnected nodes (neurons) organized in layers. Each neuron performs a weighted sum of its inputs, applies an activation function, and passes the output to the next layer [23].

ANN can learn complex patterns and capture non-linear relationships between features and the target variable. The architecture of an ANN includes the number of hidden layers, the number of neurons in each hidden layer, and the activation function used. The learning rate (alpha) controls the step size in updating the weights during training, while the regularization parameter (lambda) helps prevent overfitting [39].

Training an ANN involves forward propagation, where the inputs are passed through the network to generate predictions, and backward propagation, where the errors are propagated back to update the weights. The choice of activation function, such as sigmoid, ReLU, or tanh, affects the model’s ability to model non-linearities.

ANN can be computationally expensive, especially with large datasets and complex architectures. However, it has the advantage of being able to learn hierarchical representations of the data, making it suitable for tasks involving image recognition, natural language processing, and complex pattern recognition.

##### Naïve Bayes (NB)

Naïve Bayes is a probabilistic classifier based on Bayes’ theorem with an assumption of feature independence given the class label. Despite its simplicity, NB performs well in many classification tasks, especially when the independence assumption holds. It is fast and requires a small amount of training data [40].

NB calculates the probability of an instance belonging to a certain class by multiplying the conditional probabilities of each feature given the class. The algorithm assumes that the features are conditionally independent, which allows for efficient training and classification.

NB does not have many parameters to tune. However, it can handle both categorical and numerical features by assuming different probability distributions. For categorical features, NB uses the frequency of occurrences, while for numerical features, it applies probability density estimation [40].

One advantage of NB is its interpretability. It provides straightforward explanations of the classification decisions based on the probability calculations. NB is particularly effective in text classification tasks, such as spam filtering and sentiment analysis, where the independence assumption aligns well with the nature of textual data.

##### k-nearest neighbors’ algorithm (k-NN)

The k-nearest neighbors’ algorithm is a non-parametric, lazy learning algorithm that classifies data based on their proximity to other data points. In k-NN, the “k” represents the number of nearest neighbors used to determine the class of a given data point [41]. The algorithm assigns the class that is most common among its k nearest neighbors.

The choice of k determines the balance between bias and variance in the model. A smaller value of k (e.g., k = 1) leads to low bias but high variance, meaning the model is more susceptible to noise. On the other hand, a larger value of k (e.g., k = 5) reduces the impact of individual data points but may introduce higher bias [41].

k-NN relies on distance metrics, such as Euclidean distance or Manhattan distance, to measure the similarity between data points. The algorithm searches the training dataset to find the k nearest neighbors and assigns the class based on majority voting.

k-NN is a simple and intuitive algorithm that can be applied to both classification and regression tasks. It does not require training and is robust to noisy data. However, its main drawback is its computational complexity, especially with large datasets, as it needs to calculate distances for each query instance.

#### ML models parameters

The models’ features were categorized into two groups: 1) the dependent variable, represented by the food consumption score, and 2) the independent variables, including all associated features. The features are further represented in Table 1.

**Table 1:** The list of variables used in the machine learning model

Code	Name	Description	Value
**1**	Gender	Gender	1 = Male, 2 = Female
**2**	FCS_category	FCS Status	1 = Not Acceptable, 2 = Acceptable
**3**	FCS_variation	Variation of FCS during lockdown	1 = Decreased, 2 = The same, 3 = Increased
**4**	Region_of_living	Living region	1 = MENA, 2 = GULF
**5**	Country _Living	Arabic Countries	Bahrain, Egypt, Jordan, Palestine, Lebanon, Saudi Arabia, Emirate, Oman, Kuwait
**6**	Family_Income	Family Income Level	1 = Low, 2 = Average, 3 = High
**7**	Education	Education Level	> = Secondary, > Secondary
**8**	WatchTV	Number of Hours watching TV	1 = < One Hour, 2 = 1–2 Hours, 3 = 3–4 Hours, 5 = 5h +
**9**	Computer use	Number of Hours using computer	1 = < One Hour, 2 = 1–2 Hours, 3 = 3–4 Hours, 5 = 5h +
**10**	Employment	Employment status	1 = Employed, 2 = Unemployed
**11**	BMI	Body Mass Index (BMI)	1 = Normal, 2 = Overweight, 3 = Obese
**12**	AgeGroup	Age in years	1 = 18–23, 2 = 24–29, 3 = 30–39. 4 = 40 +
**13**	Cooking_Money	Don’t have Money for Cooking	1 = Yes, 2 = No
**14**	Access_Food	Don’t have access to food for Cooking	1 = Yes, 2 = No
**15**	Cooking_Facilities	Don’t have access to cooking tools for cooking	1 = Yes, 2 = No
**16**	Famiy_Size	Family Size	1 = < = 5 persons, 2 = > 5 Person
**17**	Financial_Problems	Financial difficulties until the end of the month	> 5 per
**18**	Financial_Shopping	Financial difficulties in Shopping	1 = Yes, 2 = No
**19**	PHA	Physical Activity during lockdown	1 = Low Active, 2 = Moderate, 3 = Highly Active
**20**	Smoking	Smoking Before COVID-19	1 = Yes, 2 = No
**21**	Mother_SHOP_3	Mother usually did food shopping	1 = Yes, 2 = No
**22**	Father_SHOP_4	Father usually did food shopping	1 = Yes, 2 = No
**23**	Hopeless	I feel hopeless	1 = Yes, 2 = No
**24**	restless	I feel restless or fidgety	1 = Yes, 2 = No
**25**	require_efforts	I feel that everything requires effort	1 = Yes, 2 = No
**26**	worthless	I feel worthless	1 = Yes, 2 = No
**27**	nervous	I feel nervous	1 = Yes, 2 = No
**28**	depressed	I feel so depressed that nothing could cheer me up	1 = Yes, 2 = No
**29**	Moretime_activity	I feel I have more time than usual in doing activities	1 = Yes, 2 = No
**30**	struggle_financially	I feel I struggle financially	1 = Yes, 2 = No
**31**	connected	I feel more connected than usual	1 = Yes, 2 = No
**32**	lockdown_duration	Lockdown duration (Weeks)	1 = < 12 weeks, 2 > = 12 weeks

The ML models were built based on the data ratio of 70:20:10 for training, testing, and validation. The grid search method and cross-validation with 10-folds were used for parameters’ optimizations. The following parameters were set for the ANN, RF, and SVM:

◦ In Artificial Neural Networks, the hidden layer had 100 neurons, with a 600-maximum number of iterations in reference to the logistic activation function.
◦ The Random Forest trees were set to 1000 with 5 maximum depth trees, and the leaf node minimum number was set to 1, while the maximum number of samples to split the internal nodes was set to 2.
◦ The SVM regularization parameter was set to 10, the RFB kernel was set to 0.001, and the bias error control factor was set to 1.

Based on the parameters optimization results, the optimized algorithms (ANN, SVM, RF) were used in identifying and predicting food insecurity.

#### Data analysis

Data cleaning, transformation, and normalization processes were conducted prior to building the ML data analysis. The final dataset consisted of 13,446 participants. The seven ML models were built and performed using the python orange data mining software, which was then used for testing and validating the ML models [42].

The study has made use of a diverse range of ML models that cover a broad spectrum of ML methodologies, from linear models (LR) to ensemble methods (GB and RF), and from instance-based methods (k-NN) to neural networks (ANN).

However, there are alternative machine learning (ML) algorithms and approaches that could potentially be explored to predict food insecurity. For example, the Decision Tree models, such as the Classification and Regression Trees (CART), which offer simplicity and ease of interpretation. Additionally, if the dataset includes images the deep learning techniques like Convolutional Neural Networks (CNN) or Recurrent Neural Networks (RNN) could also be considered. Our analysis did not include these algorithms because of the specific nature of the study problem and dataset. The utilized models were selected because of their simplicity, accuracy, and interpretability, which is crucial when addressing complex and sensitive matters like food insecurity. Deep learning models, although powerful, often function as enigmatic entities, providing limited insight into the factors contributing to their predictions. Conversely, models such as Decision Trees have the potential to overfit the training data and may not generalize well to new, unseen data. The models selected for this study aim to achieve a practical balance between predictive capability and interpretability, making them a prudent choice.

Nonetheless, it is always beneficial to explore and validate the application of other ML algorithms in predicting food insecurity. As future work, researchers could consider conducting systematic model comparison studies to identify the best performing models for this specific task. This could not only enhance the prediction accuracy but also deepen our understanding of the complex nature of food insecurity.

#### Model features importance analysis

The Shapley Additive exPlanations (SHAP) is a crucial method utilized to determine the importance of various features in interpreting the predictions made by any ML model. Drawing from cooperative game theory, SHAP equitably assigns the influence of each feature in determining the model’s outcome [21, 24]. It assists in understanding the distinct contribution of each feature to the prediction of food insecurity. This enables us to quantify the significance of each feature in the decision-making of the model.

Different performance measures were used to evaluate whether the ML models can predict food insecurity levels and the associated risk factors, such as accuracy, specificity, precision, recall, and F-measure.

## Results

### Statistical analysis

**Fig. 1:**
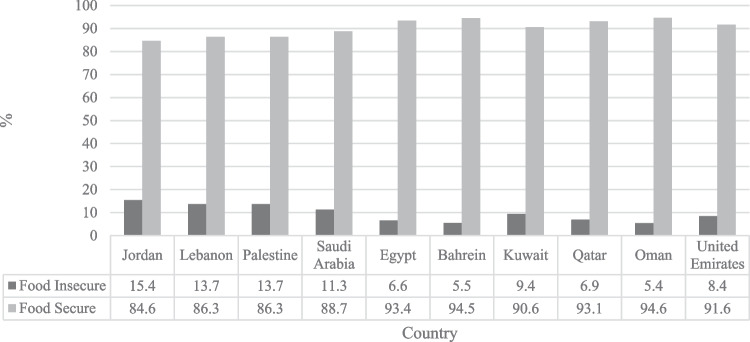
Food security status by country

The food consumption score (FCS) was used to identify the food insecure (FI) and food secure (FS) participants. The borderline and not acceptable FCS group were considered food insecure, while those with acceptable FCS were considered food secure. Overall, 9.3% out of 13,443 participants reported low and borderline FI rates among Arab countries. Results in Fig. 1 show the distribution of FI and FS by country. The findings indicate that the Jordanian, Palestinian, Lebanese, and Saudi Arabian respondents reported the highest FI rates in the region (15.4%, 13.7%, 13.7% and 11.3% respectively). On the other hand, Oman and Bahrain reported the lowest FI rates (5.4% and 5.5% respectively).

The ML models used a balanced data set of 4,259 participants extracted from the general data set of 13,443 participants. The connectivity between the associated features and food insecurity was described by the correlation matrix as illustrated in Table 2. The correlation matrix compares the target variable (FCS) with the study features and identifies which features are most correlated with the outcome variable. The results indicated that most of the study features are significantly correlated with the outcome feature except for five features: “Family Size”, “Smoking”, “I feel hopeless”, “I feel that everything requires effort”, “I feel worthless”, “I feel so depressed”, “Food shopping by mother”, “Food shopping by father”, and the “Lockdown period”. The highest correlations were found between the region and Body Mass Index (BMI) (correlation values = 0.202 and 0.143 respectively).

**Table 2:** Correlation between study features and food consumption score

Features	Correlation- Value	Features	Correlation-Value
Gender	-.090**	Use Computer	.149**
BMI	.143**	Education Level	-.057**
Region	.202**	I feel hopeless	0.017
Don’t have money for cooking	.090**	I feel restless or fidgety	.046**
Don’t have access to food for cooking facilities	.103**	I feel that everything requires effort	0.023
Don’t have access to cooking tools for cooking	.110**	I feel worthless	-0.017
Family Size	-0.029	I feel nervous	.037*
Facing Financial difficulties to the end of the month	.035*	I feel so depressed	0.012
Financial difficulties in Shopping	0.014*	I feel I have more time than usual	.045**
Physical Activity during lockdown	.075**	I feel I struggle financially	0.04*
Smoking During COVID-19	0.004	I feel more connected than usual	.063**
Food shopping by mother	0.003	Lockdown duration (Weeks)	0.015
Food shopping by father	0.005	Age (Year)	-.375**
Watch TV	.071**	Country of living during COVID19	-.139**

^*^P < 0•05, **P < 0•001

### ML models performance analysis

Table 3 depicts the performance measures in predicting participants’ FI, their accuracy, sensitivity, specificity, F1-score, and Receiver Operating Characteristic (ROC) classification curve. The accuracy score presents the crossover over the accuracy of the model, sensitivity measures the segment of FI participants correctly predicted, while specificity shows the identified segment of FS participants. According to the models’ results, the overall accuracy in predicting food insecurity ranged from 70% to 82%. Sensitivity ranged from 72% to 81.6%, while the f1-score ranged from 70.3% to 80.5%. Comparing the different ML algorithms, results indicated that GB and RF reported the highest accuracy rates (81.6% and 81.4% respectively), while the KNN algorithm had the lowest accuracy rate.

**Table 3:** FI ML prediction performance measures

#	Model	AUC (%)	ACC (%)	F1 (%)	Precision (%)	Recall (%)
**1**	Gradient Boosting	85	82	83	83	82
**2**	Random Forest	84	82	80	83	82
**3**	Logistic Regression	84	80	82	81	81
**4**	Support Vector Machine	83	80	83	81	81
**5**	Neural Network	84	80	81	81	81
**6**	Naive Bayes	80	76	77	77	77
**7**	K-nearest neighbor	72	70	71	72	72

*AUC* Area under the ROC Curve, *ACC* Accuracy, *F1* The harmonic mean of precision and recall, *Precision* The accuracy of positive predictions, *Recall* The completeness of positive predictions

**Fig. 2:**
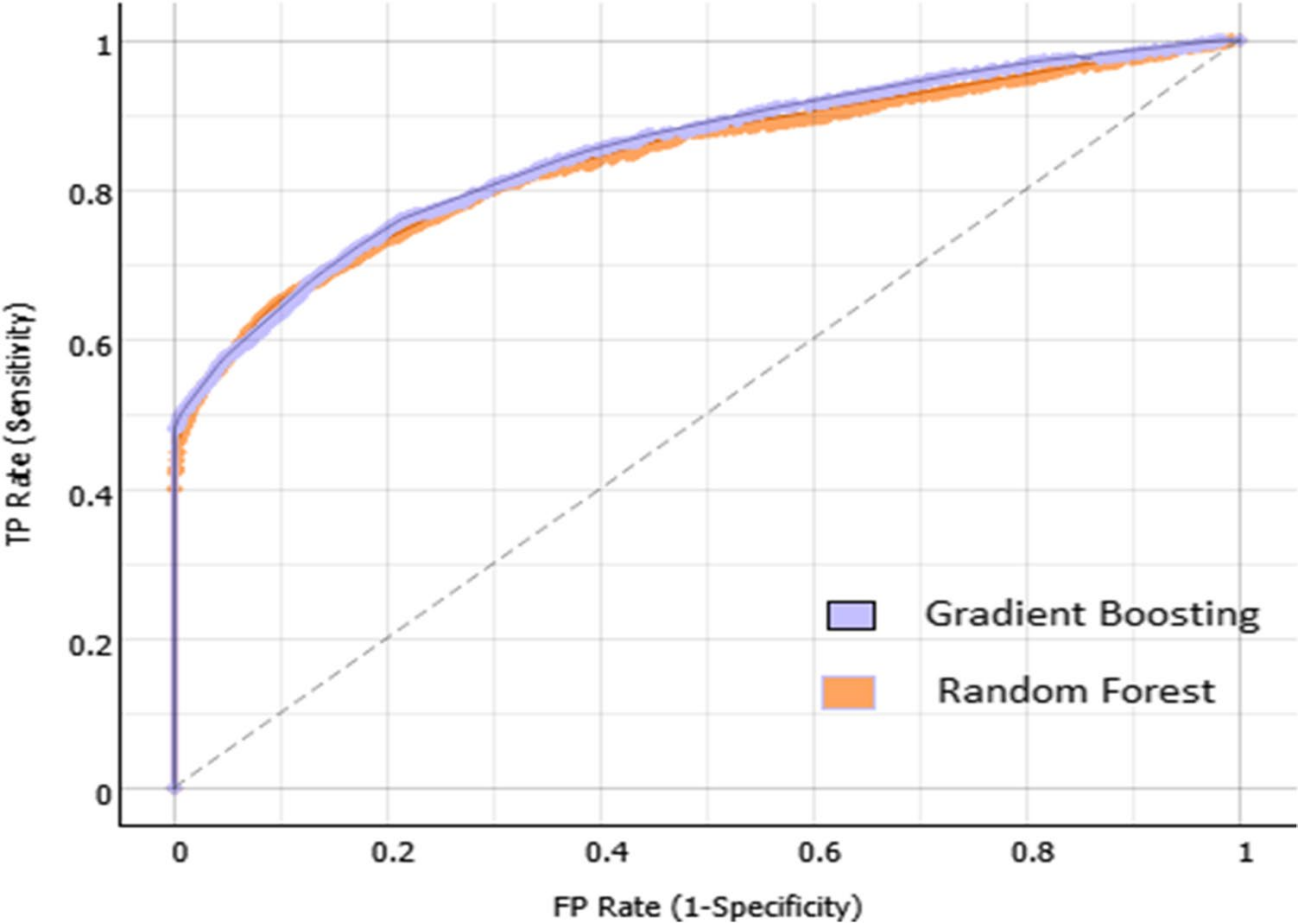
Gradient boosting and logistic regression ROC curve for below average cognitive scores (TP rate of sensitivity against FP rate of specificity)

The results in Table 3 illustrate that the algorithms’ performance is determined according to the performance measure. Thus, GB had the highest accuracy, F1-score, sensitivity, and AUC scores. However, the other algorithms reported acceptable performance measures and can be used in predicting food insecurity, except KNN and NB, which had the lowest performance levels and therefore are not recommended. The models’ performance was further evaluated using the Area Under the Curve-Receiver Operating Characteristics curve (AUC- ROC). The food consumption scores were classified into two categories: FI and FS. The ROC was obtained for the FI as indicated in Fig. 2. Results in Fig. 2 illustrate that the ROC characteristics are in the upper left side of the curve for the gradient boosting and logistic regression models, thus the two models reported the highest accuracy rates (AUC of 82%, and 80% respectively).

**Fig. 3:**
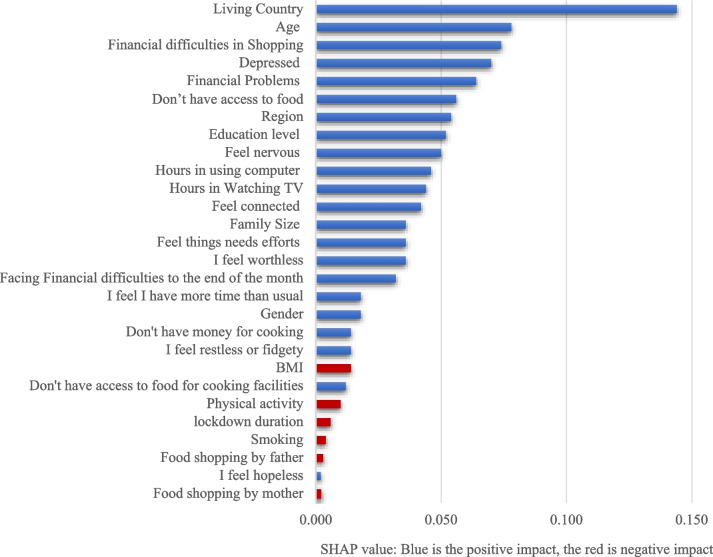
Correlation of food insecurity with the study features

The SHapley Additive exPlanations (SHAP) values analysis was used to identify the importance of study features in predicting the outcome variable and associated features. The SHAP value analysis was conducted on the GB model as it presented the best FI prediction accuracy levels. Figure 3 illustrates the correlation plot of FI participants with model predictors. The y-axis shows the FI predictors, while the x-axis shows the SHAP value. Based on the results in Fig. 3, the most important features that positively affect FI show to be (1) country of residence, (2) age, (3) financial difficulties in food shopping, (4) depression, (5) having financial problems, (6) don’t have access to food. The place of residence (living country) had the highest positive significant impact on the outcomes of FI participants. On the other hand, BMI, physical activity, smoking, food shopping by the father, and food shopping by the mother had a negative impact on the outcome of FI participants. Notably, age, depression, and feeling nervous were found to be relevant factors that play a significant role in predicting respondents’ food insecurity.

## Discussion

Food insecurity and food inequity have been increasingly relevant during and following the COVID-19 pandemic. Millions of households around the world relied on food support programs during COVID-19 [8, 43]. The lockdown-imposed work and movement limitations that significantly increased the risk of food insecurity at the global level [9, 11]. In this paper, we used ML models to predict food insecurity using a dataset containing relevant data from 10 Arab countries during the COVID-19 pandemic. The models utilized the food consumption score (FCS) as a key variable in identifying food insecurity (FI) and food secure (FS) participants as it has been used in several studies as an indicator for the prediction and identification of household FI.

This study has found that some of the features used are associated with food insecurity. Nonetheless, limited studies have used ML algorithms in identifying and predicting food insecurity, particularly in the Arab region and during the COVID-19 pandemic. The results showed significant FI levels in Arab countries, mainly in Jordan, Palestine, and Lebanon. Similarly, significantly high FI levels were evidenced in Saudi Arabia and Kuwait in the Gulf states. Furthermore, the study showed a significant relationship between the country of residence, age, and participants’ mental health status with FI levels. These findings are consistent with other similar studies that indicated a significant association between household FI and age, place of residence, and other sociodemographic variables [13, 44].

Our research identified a robust association between food insecurity and socio-economic as well as socio-demographic factors like family income, employment status, leisure time activities, educational level, sex, age, residential location, regional aspects, and mental health. These outcomes align with the conclusions drawn from other studies that explored the relationship between food insecurity and socio-demographic and socio-economic elements in Arab nations. Such studies noted that food insecurity displayed a more potent connection with life circumstances such as income level, education, gender, place of residence, housing conditions, and employment status [5, 45, 46].

Moreover, our study indicated that the Jordan, Palestine, and Lebanon populations reported the highest rates of food insecurity (FI) compared to other countries surveyed. This key finding suggests the performance of the ML model may be significantly influenced by region-specific factors, such as political, geographical and environmental, culture, and health and well-being factors. For instance, political factors, such as governmental policies, political stability, and international relations, can directly impact food availability and access in a region, thereby influencing food insecurity rates [5, 47]. Geographical and environmental factors, including climate patterns, agricultural capacity, and the vulnerability to natural disasters, can also shape the local food production and supply chains, thereby affecting the levels of food insecurity [48].

Cultural factors, such as dietary habits, attitudes towards food, and traditional agricultural practices, can significantly vary from one region to another, potentially influencing the patterns of food consumption and security [10, 43, 49]. Additionally, the overall health and well-being of a population, encompassing aspects like the prevalence of diseases, access to healthcare, and lifestyle habits, can have a direct and indirect effect on food insecurity rates [8, 48, 50].

Hence, while designing and implementing ML models for predicting food insecurity, it’s critical to account for these regional factors to enhance the model’s predictive accuracy and relevance. It’s noteworthy that food insecurity is an intricate issue with multi-dimensional influencing factors, necessitating a comprehensive, nuanced, and context-aware approach in its prediction and management.

Interestingly, we found that BMI had a strong correlation and association with FI. The negative association between BMI and FCS indicated that food-insecure people had higher BMI scores. This finding was consistent with other similar studies [10, 51].

The implementation of the ML models demonstrates the power of ML in predicting FI from the associated factors. The performance analysis included the accuracy, recall, precision, F1-score, and ROC measures. The results showed that GB and RF are among the highest-performing ML models in predicting FI. However, the other employed models showed an acceptable performance rate, while KNN reported the lowest accuracy rate of all. Our results were found to be consistent with other studies that used ML in predicting FI and confirmed the feasibility of ML use in identifying FI [24, 52, 53]. The models in Doreswamy et al. study reported accuracy rates ranging from 70% to 82%, while other models reviewed from previous research reported accuracy rates ranging from 55% to 85% [29]. Other studies found that RF and SVM had high performance rates in predicting FI [24, 53]. Furthermore, the ML models identified a significant association between mental health factors and FI outcomes, including depression, stress, hopelessness, and negative feelings. The findings were consistent with other studies that showed a significant association between FI outcomes and depression, anxiety, despair, and hopelessness [31, 54].

The study used SHapley Additive exPlanations (SHAP) values to interpret the ML models. SHAP is a unified measure of feature importance that assigns each feature an importance score for a particular prediction. These values are calculated in such a way that they allow fair distribution of the contribution of each feature to the prediction for each individual instance in the data. It’s a game-theoretic approach to explain the output of any ML model [24, 53].

In the context of our study, it appears that country of residence, age, financial difficulties in shopping, depression, inaccessibility to food, and financial problems were the most crucial factors influencing the ML model outcomes. This implies that these are the key contributors to food insecurity as per the ML model’s analysis.

Interestingly, the findings were in line with other studies indicating that food prices, poor access to food and markets, poverty, living place, and lack of education were associated with food insecurity. This validates the model’s outcomes, enhancing the confidence in the identified critical features. It shows that, despite the complexity of the ML models, the results correspond to those of more conventional research methods, thereby supporting the robustness and reliability of the models. Among the most notable findings was the prominence of mental health factors in predicting food insecurity. Depression and nervous feelings were among the most important factors associated with food insecurity outcomes. The importance of age in the model suggests that food insecurity impacts different age groups disproportionately. These insights could be instrumental in driving policies and interventions to mitigate food insecurity by focusing on these key identified areas. Our findings were consistent with other studies indicating that food prices, poor access to food and market, poverty, living place, and lack of education were associated with food insecurity. Notably, depression, nervous feelings and age were among the most important factors associated with FI outcomes [5, 10, 12, 14].

Moreover, the presence of financial difficulties and inaccessibility to food highlight the role of socio-economic factors in food insecurity. These could be areas of focus for policymakers and could guide strategies to reduce economic barriers to food access. The study shows how SHAP values can be effectively used to enhance interpretability in complex ML models, providing practical insights into the specific factors contributing to food insecurity.

The pandemic encouraged the consideration of food access equity at the global level, mainly with regards to ensuring the availability of basic food, housing, health, and education. Our approach introduced an important contribution to improving the currently available methods of predicting and early warning of food insecurity as it has proven effective in identifying and predicting food insecure people from food consumption levels. The development of this model was based on international data from Arab countries to ensure its replicability at the global level, among marginalized and conflict-prone areas.

Our research findings hold considerable implications for stakeholders aiming to design targeted interventions to mitigate food insecurity. Leveraging ML techniques has allowed the identification of significant predictors associated with food insecurity, namely: country of residence, age, financial stability, access to food, and mental health conditions, specifically depression. This key understanding will assist policy makers in designing more precise, effective strategies.

The association of food insecurity with country of residence suggests the existence of regional differences. These may arise from a complex mixture of socio-economic factors like employment opportunities, income levels, and infrastructure. This necessitates the need for policymakers to focus on region-specific initiatives to mitigate food insecurity. The initiatives may include the creation of employment opportunities, improving infrastructure for better access to food, and devising regional food aid programs. The study indicated that age is a significant predictor means that different age groups experience varying levels of food insecurity. This insight should prompt policymakers to create age-focused policies to address the unique needs of each demographic.

Furthermore, the close linkage between financial instability, difficulties in accessing food, and food insecurity underscores the critical role of economic stability in ensuring food security. This observation requires interventions focusing on enhancing economic stability, such as poverty alleviation initiatives and steps to improve food accessibility and affordability. The fact that depression was found to be an important predictor of food insecurity highlights the need to integrate mental health support within food aid programs, providing a holistic approach to combating food insecurity.

Moreover, the use of ML models in identifying the predictors of food insecurity can significantly aid policymakers in creating targeted and nuanced interventions.

### Model deployment ethical considerations

The use of ML to predict food insecurity indeed provides promising results, but it is accompanied by ethical concerns that need careful attention. Data privacy and security stand as critical considerations. Given that ML operates on extensive datasets, which may sometimes encompass sensitive information, issues related to data acquisition, anonymization, and usage are of paramount importance. It is necessary that privacy of the individuals whose data is utilized is assured, and data protection standards are rigorously upheld. It is also worth noting that bias and fairness in both data and ML models need to be addressed. Furthermore, the interventions that are developed based on the predictions from these models could inadvertently stigmatize or isolate certain groups in society, an outcome that is contrary to the intentions of such initiatives.

#### Strengths and limitations

The pandemic calls for a shift in thinking and consideration of access to food and ensuring food security among low- and middle-income countries. This model was designed to respond to the need for early detection of food insecurity to ensure rapid and accessible humanitarian response. The model provides a precise and accurate FI identification and prediction tool that is less dependent on traditional assessment and analysis. Thus, the model improves automatic and early detection of food insecurity, which in turn can enhance rapid intervention and policy-making programs for combatting food insecurity. Thus, this research study not only introduces ML techniques in predicting FI during and after pandemic conditions, but also enhances data driven decision making and early intervention.

Potential limitations in this study could stem from various types of bias within the data, such as sampling bias. The data set were extracted from the Corona Cooking Survey which doesn’t reflect the socio-economic, cultural, geographical, and age diversity across the ten Arab countries, it could result in selection bias, potentially affecting the model’s predictive accuracy. Moreover, measurement bias from self-reported data could also influence the study, as inaccuracies or inconsistencies in responses can affect the model’s precision. While the cross-validation with 10 folds modeling approach was used for avoiding model overfitting.

Also, the study’s feature selection could present a limitation. Even though 32 features were used to predict food insecurity, vital factors like political factors, social support, or individual health status might not have been accounted for. Hence, while the model offers valuable insights, it might not completely encapsulate the complexity of food insecurity. Finally, the unique circumstances of the COVID-19 pandemic might have influenced the findings, which may not align with typical conditions, suggesting potential variances in the model’s performance under normal circumstances.

Nonetheless, the study was limited by the number of features used in predicting FI. In some countries, the model data set presented a lower number of participants, which limited the ability of building ML models per country. The research findings encourage future research by building more complex ML models that might improve prediction and classifications accuracy.

## Conclusions

The study assessed the performance measures of seven ML algorithms in predicting the risk factors associated with food insecurity during the COVID-19 pandemic. The research found that Gradient Boosting and Random Forest are among the highest-performing ML algorithms for the accurate prediction of food insecurity. The study developed an ML model that can enhance the early detection of FI, and that can be replicated in other regions. The study contributes to the literature on food insecurity based on FCS by utilizing the ML methods in identifying the key characteristics of food insecurity in the Arab region, to determine the relationship between the food consumption score and the associated factors, and to support policy makers with advanced food insecurity identification and prediction tools. Furthermore, the use of ML models is a valuable tool in improving food insecurity prediction and detection over time, with enhanced granularity, with the ability to share data, to incorporate wide range of variables, and to make use of automation for effective prevention and intervention programs at the regional and individual levels.

## Data Availability

The datasets generated and/or analysed during the current study are available in the Corona Survey repository, https://osf.io/nz9xf/files/.

## Abbreviations

FI: Food insecurity
SOFI: State of Food Insecurity and Nutrition in the World
COVID-19: Coronavirus disease 2019
ESCWA: Social Commission for Western Asia
WFP: World Food Program
GCC: Gulf Cooperation Council
FCS: Food Consumption Score
ML: Machine Learning
KNN: K-Nearest Neighbor
RF: Random Forest
SVM: Support Vector Machine
LR: Logistic Regression
NB: Naïve Bayes
ANN: Artificial Neural Network
FS: Food Secure
BMI: Body Mass Index
ROC: Receiver Operating Characteristics
AUC: Area under the curve
